# Characterization of Chemically Activated Carbons Prepared from Miscanthus and Switchgrass Biomass

**DOI:** 10.3390/ma13071654

**Published:** 2020-04-02

**Authors:** Beata Doczekalska, Monika Bartkowiak, Bogusława Waliszewska, Grażyna Orszulak, Joanna Cerazy-Waliszewska, Tomasz Pniewski

**Affiliations:** 1 Institute of Chemical Wood Technology, Poznań University of Life Sciences, Wojska Polskiego 38/42, 60-637 Poznań, Poland; monika.bartkowiak@up.poznan.pl (M.B.); bwaliszewska@up.poznan.pl (B.W.); grazyna.orszulak@gmail.com (G.O.); 2 Institute of Plant Genetics of the Polish Academic of Sciences, Strzeszyńska 34, 60-479 Poznań, Poland; jcer@igr.poznan.pl

**Keywords:** activated carbon, miscanthus, switchgrass, porosity

## Abstract

Lignocellulosic biomass, including that of energy crops, can be an alternative source to produce activated carbons (ACs). Miscanthus and switchgrass straw were used to produce ACs in a two-step process. Crushed plant material was carbonized at 600 °C and then obtained carbon was activated using NaOH or KOH at 750 °C. The content of surface oxygen groups was determined using Boehm’s method. The porosity of ACs was assayed using the nitrogen adsorption/desorption technique, while their thermal resistance using the thermogravimetric method. The ACs derived from miscanthus and switchgrass were characterized by surfaces rich in chemical groups and a highly developed porous structure. The highest specific surface areas, over 1600 m^2^/g, were obtained after carbon treatment with NaOH. High values of iodine number, 1200–1240 mg/g, indicate an extensive system of micropores and their good adsorption properties. The type of activator affected the contents of oxygen functional groups and some porosity parameters as well as thermal stability ranges of the ACs. Among obtained carbons, the highest quality was found for these derived from *M. sacchariflorus* followed by switchgrass, after activation with NaOH. Hence, while these crop species are not as effective biomass sources as other energy grasses, they can become valuable feedstocks for ACs.

## 1. Introduction

Since the moment activated carbon (AC) was obtained for the first time in 1900 by Raphael Von Ostrejko, considered to be the “father of activated carbon”, the demand for this product has been permanently growing [1]. The market for AC is expected to register a Compound Annual Growth Rate of 6.31% during the forecast period of 2019–2024. Major factors driving the market studied include conformance to stringent environmental regulations in water treatment applications and increasing the importance of air pollution control, especially mercury removal [2]. Thanks to its high adsorption capacity and large surface area, AC dominates on the global adsorbent market, accounting for over 65% of the market trade volume [3]. AC has become the preferred option for use in potable water purification, treatment of aquariums, swimming pools and wastewater as well as air and gas filtration. These applications are the most important due to increasing environmental pollution, health concerns and stringent government regulations [4]. Apart from that, AC is used in the food industry for the decolorization and deodorization of food and beverages, and in the pharmaceutical industry for the purification of vitamins, antibiotics and other compounds [5]. Other applications of activated carbon include monitoring gas emissions in automobiles, personal protection in the defense sector, recovery of gold and precious metals and as a catalyst in the removal of mercaptans in oil refineries [6,7]. ACs are also increasingly popular in electrochemistry, for energy storage and production of capacitors and Li-ion batteries [8,9].

The AC market is currently facing price issues due to the shortage of resources and increasing concerns concerning the supply chain. However, the market is on the rise due to the extensive use of AC in applications for the liquid and gas phase [3]. Carbon porous materials may be obtained from many fossil and organic feedstocks. Initially, coal, lignite, crude oil and wood were most frequently used for the production of porous materials. Over time, the activated carbon industry was forced to look for alternative precursors that would be cheaper and meet environmental requirements in view of increased costs of traditional feedstocks and severe fines for environmental pollution. Lignocellulosic biomass has proved to be an attractive solution to these problems from both economic and ecological points of view. Many types of agricultural and forestry waste materials as straw, fruit stones, shells of walnuts, pistachios and coconuts, cones, branches and carps, as well as seaweed, have been exploited to produce ACs [10,11]. Thanks to carbonization and activation, these wastes can be converted into valuable porous materials instead of undergoing gradual degradation and useless loss [11,12].

Nevertheless, energy crops have been investigated for several years as an alternative feedstock for carbonization. Thanks to the fast and abundant biomass production, they represent a considerable resource. Such grasses as particularly miscanthus species (*Miscanthus* spp.) or switchgrass (*Panicum virgatum*) may provide demanded amounts of raw material thanks to their adaptability to various environments, especially in countries of temperate climate [13,14,15]. Depending on the species and local cultivation conditions, the biomass production for miscanthus ranges from 9.0 to 30 t/ha, the lowest being for *M. sacchariflorus* and the highest for *M.* × *giganteus*, while the yield range for switchgrass is 6–20 t/ha [16,17,18,19]. The global cultivation area of these crops may be easily expanded, since these grasses are able to grow on marginal lands [20,21,22]. In fact, miscanthus and switchgrass biomass have already been grown to produce biochar of potential use for soil and groundwater remediation or improvement [23,24,25,26]. However, making ACs for industrial or medicinal and other applications requires additional processing of crude carbonizate and the evaluation of obtained products. So far, relatively few studies have been conducted on the subject regarding *M.* × *giganteus* [9,27].

The objective of this study was to prepare activated carbons from the biomass of energy grasses, *Miscanthus* × *giganteus*, *M. sinensis*, *M. sacchariflorus* and *Panicum virgatum*, and evaluate their porosity. Crude carbonizates were activated chemically using KOH and NaOH. Physical and chemical properties of the obtained activated carbons, including surface chemical groups, porosity parameters and thermal stability, were characterized.

## 2. Materials and Methods 

### 2.1. Plant Materials

All genotypes of miscanthus (*Miscanthus* spp.) and switchgrass (*Panicum virgatum*) used as the feedstock in the study were received from the collection of TINPLANT GmbH (Klein Wanzleben, Germany). *Miscanthus* × *giganteus* was represented by the M×g3 ecotype, i.e., genotype M116—a synthetic hybrid obtained by TINPLANT GmbH—and then cultivated for several seasons at the Novosibirsk State Agriculture University (Russia). In turn, *M. sinensis* was represented by genotype 93M0006004 of Japanese origin, while *M. sacchariflorus* was represented by the Chinese diploid genotype 96M0003. Switchgrass was represented by the diploid genotype Kanlow. All miscanthus plants were obtained from rhizomes, while switchgrass from seeds. The plants have been cultivated since 2009 on medium-fertile Orthic Luvisol soil (FAO classification) in the field located at the Institute of Plant Genetics, Polish Academy of Sciences (IPG PAS) in Poznań (52°25′ N, 16°55′ E) [19]. No fertilizers were applied during the entire cultivation period. Weather conditions were typical of west-central Poland [28], characterized by locally low precipitation (500–600 mm yearly). For the study, plant biomass was harvested in winter 2017, then chopped and stored in a cold and dry place until used.

### 2.2. Analysis of Biomass Composition

Particular components of biomass (dry matter (DM)) were assayed according to the TAPPI standards, including: (a) cellulose by Seifert’s method using an acetylacetone-dioxane mixture [29]; (b) holocellulose using sodium chlorite (TAPPI—T 9 wd-75); (c) pentosans by Tollens’ method using phloroglucinol (TAPPI—T 233 cm-84); (d) lignin by the TAPPI method using concentrated sulfuric acid (TAPPI—T 222 om-06); (e) substances soluble in organic solvents according to Soxhlet (TAPPI—T 204 cm-07); (f) substances soluble in cold and hot water (TAPPI—T 207 om-88); (g) substances soluble in 1% NaOH (TAPPI—T 212 om-07); and (h) ash (TAPPI—T 211 cm-86). Hemicellulose theoretical content was calculated as the difference between holocellulose and cellulose.

### 2.3. Preparation of Activated Carbon

The lignocellulosic materials were ground using an SM100 mill (Retsch GmbH, Haan, Germany) equipped with a 0.5 mm sieve and next carbonized. This process was carried out in a chamber reactor in the oxygen-free atmosphere by heating to 600 °C at a rate of 3 °C/min and then maintained under stable conditions for 1 h. Carbonizates after grinding in mortar were activated with potassium or sodium hydroxide at the 1:4 ratio (w/w) in the argon atmosphere at 750 °C for 15 min in a non-porous ceramic reactor (Czylok, Jastrzębie-Zdrój, Poland). Activated carbons (ACs) were washed out with 1% hydrochloric acid and then deionized water to the neutral pH.

### 2.4. Characterization of Activated Carbons

Contents of surface oxygen groups (mmol/g) were determined according to Boehm’s method [30]. Briefly, 4 samples of activated carbon, 250 mg each, were placed separately in 250 mL flasks. Then, each sample was supplemented with 25 mL of 0.1 M NaOH, 0.1 M NaHCO_3_ or 0.05 M Na_2_CO_3_ (to assay acidic groups) or 0.1 M HCl (to assay basic groups), and the mixtures were shaken at ~120 rpm for 24 h at room temperature. After filtering the mixtures, 10 mL of each filtrate was pipetted and the excess of bases and acids was titrated (Tashiro indicator) using 0.1 M HCl or NaOH, respectively. All assays were repeated three times. Numbers of acidic sites of various types were calculated according to the formula below under the assumption that NaOH neutralizes carboxyl, phenolic and lactonic groups: Na_2_CO_3_, carboxyl and lactonic ones, and NaHCO_3_, only carboxyl groups. The number of surface basic sites was calculated according to the same formula, but this time from the amount of HCl which reacted with carbon.

(1) $$G_{x}=\left(V_{0}-V_{x}\right)\times c\times \frac{25}{W_{x}}\left[\frac{\mathrm{mmol}}{g}\right]$$

where:

*G_x_*—content of functional groups of a given type;
*V*_0_, *V_x_*—volumes of NaOH or HCl solutions (for assays of acidic or basic groups, respectively) used for titration of assayed (*V_x_*) and blank samples (*V*_0_) (mL);
*c*—HCl or NaOH concentration (M);
*W_x_*—weight of carbonizate sample (g).

The specific surface area and pore size distribution were determined by analysis of nitrogen adsorption at −196 °C (ASAP™2020, Micromeritics Instrument Corp., Norcross, GA, USA). Samples before measurement were degassed at 300 °C for 10 h at a pressure of 10^−6^ Pa. Collected sorption data facilitated calculation of the following structural parameters in the area of micro- and mesopores:

S_BET_—specific surface area (m^2^/g)—by the BET method, to the relative pressure p/p_0_ ≈ 0.2;
V_T_—a total pore volume (cm^3^/g)—determined from the isotherm at a relative pressure p/p_0_ ≈ 0.975;
V_meso_—mesopore volume by the BJH method (cm^3^/g);
V_micro_—micropore volume from differences V_T_ − V_meso_ (cm^3^/g);
d_av_—average pore diameter calculated using the formula d_av_ = 4V_T_/S_BET_ (nm).

Iodine numbers (IN) of activated carbons were determined on the basis of the Standard Test Method ASTM Designation: D 1510-57 T ASTM-D4607-94. Thus, iodine number (mg I_2_/g carbon) was measured by titration at 30 °C. This parameter indicated the extent of micropore distribution in activated carbon and allowed evaluating its adsorption capacity. From each activated carbon, three dried samples (0.1 g) were placed into separated flasks and fully wetted with 10 mL of 5% HCl. Then 100 mL of 0.025 M standard iodine solution was poured into the flask and the content was vigorously shaken for 30 s. After quick filtration, 50 mL of the solution was titrated using 0.1 M sodium thiosulfate with starch as an indicator. The concentration of iodine in the solution was calculated according to the formula below from the total volume of sodium thiosulfate used.

(2) $$\mathrm{IN}=\frac{\left(V_{0}-V_{x}\right)\times c_{thio}\times 126.92}{m}\left[\frac{\mathrm{mg}}{g}\right]$$

where:

*V*_0_, *V_x_*—volumes of sodium thiosulfate solution used for titration of assayed (*V_x_*) and blank samples (*V*_0_) (mL);
*c_thio_*—concentration of sodium thiosulfate solution (M);
*m*—activated carbon sample (g);
126.92—mass of 1 mole of iodine (g).

Thermogravimetric (TG) analysis of activated carbons was carried out on a LabsysTM thermobalance (Setaram Instrumentation, Caluire, France) under the following conditions: final temperature 1200 °C, rate of temperature increase at 5 °C/min and helium atmosphere at the flowing rate of about 2 dm^3^/h. Mass loss of a sample was calculated in %.

In order to facilitate the identification of activated carbons, the sample description system was adopted as shown in Table 1.

### 2.5. Statistical Analysis

Obtained data were analyzed by two-way ANOVA (biomass composition, content of surface oxygen functional groups in activated carbons) followed by the post-hoc Tukey’s test or one-way ANOVA (parameters of the porous structure and thermal stability of ACs). Statistical analysis was performed using the Statistica 13.0 statistical software package (StatSoft Inc, Tulsa, OK, USA). 

## 3. Results

The chemical composition of biomass, as the feedstock to produce activated carbon, was assayed first (Table 2 and Table 3). The content of holocellulose, comprising all carbohydrate components, was almost identical in biomass of individual miscanthus species, while that for switchgrass was significantly lower. Correspondingly, the content of cellulose, the major part of holocellulose, was similar in all three miscanthus species, while it was significantly higher than in switchgrass. In contrast to cellulose, the contents of hemicellulose and its predominant part, i.e., pentosans, did not differ between miscanthus species and switchgrass. In comparison to the observable tendencies in polysaccharide contents, that for lignin did not reflect biological classification. Significantly the most lignified was biomass of MG, followed by MSac and SG, whereas MSin was characterized by the lowest extent of lignification. However, the cellulose-lignin ratio only partially corresponded to lignin content. The highest ratio was recorded for MSin, medium for MSac, while it was the lowest for MG and SG (Table 2).

Contents of all categories of extractives were significantly higher in switchgrass in comparison to miscanthus. This discrepancy was particularly evident for substances soluble in cold or hot water. However, some differentiation was also observed among the miscanthus species. Except for substances soluble in cold water, contents of the other components in the MG biomass were closer to the lower limit of the range. Generally, MSin contained more extractives, while their levels in MSac varied, depending on the category. In turn, ash content was significantly lower in MSac and SG compared to MG and MSin (Table 3).

Despite the differentiation of biomass chemical composition, its impact on the physicochemical properties of activated carbons was secondary in comparison to an activator (Table 4, Table 5 and Table 6). Regardless of the type of precursor and hydroxide used at the activation temperature of 750 °C, significantly more acidic groups were formed, 1.1–2.6 times compared to basic groups (Table 4). The former ones constituted approx. 70% in the case of KOH activation and approx. 60% in the case of NaOH activation, and this advantage was significant in both cases. Phenolic groups had the largest share in the total acidity of the surface and only for this category the effect of the activator was not evident and was partially modified by the precursor. However, in the case of lactonic and carboxyl groups, for all precursors, their content was significantly higher when KOH was used as an activator. The change of an activator from KOH to NaOH caused a significant increase in the number of basic oxygen groups, regardless of the type of precursor.

**Figure 1:**
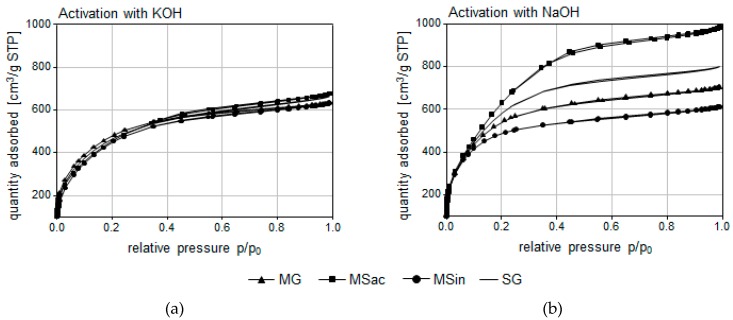
Isotherms of N_2_ adsorption–desorption by ACs obtained from biomass of miscanthus species and switchgrass using KOH (**a**) or NaOH (**b**) as the activator.

Similarly to the chemical nature of the surface, the effect of an activator was also evident regarding parameters of porosity, whereas the type of precursor was less important (Table 5). Specific surface area and volume of micro- and mesopores in produced ACs were calculated on the basis of sorption data from nitrogen adsorption/desorption isotherms at −196 °C (Figure 1).

According to the IUPAC classification, the isotherms of nitrogen adsorption-desorption in analyzed ACs were of type I. Steep sorption curves in the range of very low relative pressures (p/p_0_ < 0.1) up to nitrogen sorption volumes close to 400 cm^3^/g STP indicated a well-developed system of micropores. All obtained ACs were characterized by extensive specific surface areas and large total pore volume. However, evidently higher S_BET_ values and larger volumes of micropores were obtained for ACs activated with the use of NaOH, where the effect of activator was significant. The V_T_ values were also higher for most ACs obtained by NaOH activation, yet here, this effect was statistically non-significant. The mesopore volumes differed among ACs activated with NaOH, while they were almost identical in the case of KOH. Low variability was also found for values of pore width and IN, although for most precursors they were slightly higher for ACs obtained by NaOH activation compared to KOH. In summary, it can be said that the most developed porous structure, especially regarding such parameters as S_BET_, V_micro_ and V_meso_, and IN, was found in ACs MSac/NaOH, followed by SG/NaOH (Table 5).

The results of TG analysis (Table 6) indicate varied thermal properties of the obtained carbon materials. However, analogously to the surface chemical groups and parameters of a porous structure, activators also had a greater effect than precursors, as indicated by respective p-values for particular temperature ranges. The effect of activator was especially evident for the temperatures from 500 to 900 °C, where it was statistically significant (Table 6). Relative mass losses demonstrated that ACs obtained by NaOH activation were more stable, except for the highest temperatures only. Nonetheless, the total mass losses determined for the whole measurement range (20–1200 °C) showed comparable thermal stability of ACs in respect of an activator. All in all, the ACs MG/NaOH, MSin/NaOH and MSac/KOH may be considered the most thermally resistant.

## 4. Discussion

### 4.1. Composition of Miscanthus and Switchgrass Biomass as a Feedstock for Carbonization

Porous structure and other characteristics of activated carbon are determined by the properties of the feedstock and conditions of carbonization and activation. In turn, the chemical composition of biomass depends on many factors such as plant species, type of organ and tissue, growth conditions including climate, year of cultivation and harvesting time, etc. [31]. In the study, the winter-harvested biomass was used, as it is usually used for practical purposes and also contains higher levels of structural biopolymers, mainly holocellulose and lignin.

Here, holocellulose content in miscanthus corresponded with data recorded for plantations in the temperate climate zone [19,32,33]. Lower holocellulose contents in switchgrass biomass were also observed earlier [34,35]. Regarding cellulose, the main holocellulose component, the determined contents for *M.* × *giganteus*, *M. sacchariflorus* and *M. sinensis* (Table 2), also fell within the ranges for these species cultivated in other temperate regions [19,32,33,36,37]. In turn, Gismatulina and Budaeva [38] reported higher cellulose contents in biomass of *M. sinensis*, but cultivated under the severe continental climate of Siberia. In comparison to miscanthus, cellulose content in switchgrass was significantly lower (Table 2), which corresponds to data reported by other authors [18,33,34,35]. 

Unlike cellulose, the hemicellulose fraction in the tested raw materials did not differ significantly (Table 2). Yet, a slightly higher hemicellulose content was found in the species characterized by a lower cellulose fraction. Again, the observed amount of these low-polymerized carbohydrates corresponded with data reported for miscanthus and switchgrass cultivated in the temperate climate [32,33,34,35,36,37]. The content of pentosans corresponded to that for hemicellulose, as these glycans constitute a major fraction of the latter. Analogously, their content did not vary between the tested materials, similarly to an earlier report [19]. A larger discrepancy in the content of pentosans, between genotypes and also plant parts, was reported only for *M. sinensis* cultivated in the continental climate [38,39].

In contrast to hemicellulose, lignin content varied between the investigated grasses; yet this differentiation did not correspond to their genera as it was the case with cellulose. Biomass of *M.* × *giganteus* contained significantly the highest amount of lignin, while in the *M. sinensis* biomass it was the lowest (Table 2). This range of values is approx. 1.5–2 times higher in comparison to data from regions of maritime or warmer climates [33,36,37], while it was similar to that for miscanthus or switchgrass cultivated under the continental climate [19,33,35,38]. Interestingly, the biomass of *M. sacchariflorus* and switchgrass, characterized by an intermediate lignin content, slightly above 20%, after processing yielded ACs of the most developed porous structure (Table 5). However, this cannot be related to the cellulose-lignin ratio, as this parameter is determined by two other traits. 

In contrast to structural cell wall biopolymers, switchgrass DM biomass, in comparison with miscanthus, contained more extractives, which include free sugars, proteins, dyes, waxes and other soluble compounds (Table 3). In particular, substances soluble in cold and hot water, were much more abundant, but the contents of other extractives were also significantly higher in switchgrass. Our results are comparable with data from previous reports, mostly regarding miscanthus and switchgrass coming from the transitional or continental climate [19,32,34,36,38,40]. 

Compared to woody biomass, grasses are typically characterized by a higher content of ash, i.e., mineral compounds [41]. Low ash content (Table 3) in the tested miscanthus species was comparable to previously reported lower contents (<3%) of mineral compounds in miscanthus biomass [19,32,33,42]. In the case of switchgrass, determined ash content can be classified as low in comparison to the reported 2.1%–8.8% [18,35]. However, most authors reported higher ash contents, showing a considerable effect of growing conditions, including year of cultivation, location, type of soil or fertilization [18,33,35,37,38,39,40,42,43,44]. In this study, the biomass of *M. sacchariflorus* and switchgrass contained significantly lower amounts of ash than the other miscanthus species. It cannot be excluded that a low content of mineral compounds was reflected in the formation of a more developed porous structure in ACs (Table 5).

### 4.2. Properties of Activated Carbons Derived from Miscanthus and Switchgrass Biomass

The biomass of all tested energy grasses was carbonized at 600 °C according to the commonly adopted technique. All ACs derived from crude carbonizates were characterized by a rich chemical structure of the surface and substantial porosity (Table 4 and Table 5). The effect of used activators, i.e., KOH or NaOH, was much more evident than that of the precursor and it was statistically significant in the case of some AC parameters. Regardless of the type of precursor and hydroxide used, mainly acidic groups were generated (Table 4). They were predominantly phenolic groups, followed by lactonic and carboxyl groups. However, for all precursors, significantly more groups of the two latter types were identified on the surface of the carbons obtained after activation with KOH. When NaOH was used as the activator, the number of basic oxygen groups significantly increased. Hence, based on the obtained results of the Boehm analysis, it may be concluded that the mechanisms of KOH and NaOH action on carbon precursors are not identical, which directly affects the surface structure of carbon materials.

The mechanisms of KOH and NaOH action on carbonizate precursors has been described (see chemical Equations (3)–(10) below) by many scientists [45,46,47,48,49,50]. It has been found that hydrogen is the product of the reaction for both these hydroxides with a carbon matrix (Equations (3) and (4), where Me is a potassium or sodium ion) [48,50]:

4MeOH + −CH_2_→Me_2_CO_3_ + Me_2_O + 3H_2_ (3)

6MeOH + 2C→2Me + 3H_2_ + 2Me_2_CO_3_ (4)

The reaction of the alkaline activator within the temperature range of 400–700 °C leads to increased CO_2_ production (Equations (5) and (6)) [47,50]:

Me_2_CO_3_→Me_2_O + CO_2_ (5)

4MeOH + C→4Me + CO_2_ + H_2_O (6)

During the heating of carbons also the CO emission occurs. Carbon monoxide is the product of carbon with sodium or potassium oxides and carbonates (Equations (7) and (8)) [50].

Me_2_O + C→2Me + CO (7)

Me_2_CO_3_ + C→2Me + 3CO (8)

In turn, the release of water vapor is primarily associated with the physical state of the activators used, as well as the physicochemical properties of hydroxides. The course of water release curves is different. In the carbon activation process using KOH, water is released at temperatures from 300 to 500 °C, whereas with the use of NaOH it is only above 600 °C. It is assumed that metallic hydroxide activators react with carbon, volatile oxidation products or decompose with the release of water (Equations (6), (9) and (10)) [47,50].

4MeOH + 2CO_2_→2Me_2_CO_3_ + 2H_2_O (9)

2MeOH→Me_2_O + H_2_O (10)

Activation with NaOH leads to the release of larger volumes of reaction gases at higher temperatures than when activated with KOH. Thus, above 600 °C, Equations (6) and (9), leading to the release of water vapor and carbon dioxide, are more likely for carbon activation with NaOH. Additionally, the emission of hydrogen generated in the reaction of NaOH with a carbon matrix appears in the reaction space also at temperatures higher than at the application of KOH. This may indicate a lower share of sodium carbonate in activation processes than in the case of potassium carbonate, which is formed at higher temperatures of carbon activation using KOH. At temperatures above 700 °C, the emission of carbon monoxide is evidently more intensive when NaOH is used [48,49]. On this basis, it can be concluded that the formation of acid surface groups is influenced not only by the oxygen content in carbonizate, but also by the amount of oxygen supplied from products of the reaction between an activator and carbon. Having in mind the thermal stability of individual functional groups, it should be stated that lower concentrations of acid groups in carbons obtained after NaOH activation are associated with more difficult oxidation of the carbon surface than in the case of KOH activation.

All ACs obtained from miscanthus and switchgrass biomass were characterized by a developed porous structure, as indicated by the high iodine numbers and direct assays (Table 5). As IN is an indicator of microporosity (pores < 1 nm in diameter), higher INs reflect better development of the microporous structure, on which greater adsorption abilities for low-molar-mass solutes depend to a large extent [51]. This should definitely be a subject of the next phase of research. At this moment, it can be stated that an extensive specific surface area and large pore volume were found for virtually all ACs. However, higher values, especially of S_BET_, V_micro_ (both also with the determined significant effect of activator), as well as V_T_ and IN, were found for ACs obtained after the use of NaOH. Thus, analyses of AC porosity also confirmed that the mechanism of action for KOH and NaOH hydroxides is different.

Structural and surface parameters were similar for all ACs, yet the best were found for MSac/NaOH followed by SG/NaOH, i.e., those obtained by NaOH activation of carbonizates from *M. sacchariflorus* or switchgrass biomass, respectively (Table 5). It is difficult to comprehensively explain the reason for this fact, especially since the chemical composition of all used biomass feedstocks was similar (Table 2 and Table 3). However, certain selected common characteristics of MSac and SG, significantly different from the other feedstocks, may be indicated. These were the above-mentioned intermediate lignin contents and low amounts of ash, which synergistically might have exerted some positive impact on the porous structure of obtained ACs. This possible effect, expanded to cover the lignin subunit composition and ultrastructure, could be a subject of further detailed research. Literature studies also indicate that each natural material requires an individual approach and the mechanism of pyrolysis is specific to a particular raw material. It is related to the diversity in the structure of lignocellulosic biomass, i.e., different chemical composition and diverse anatomical structure [52].

The values of S_BET_ and other parameters of porous structure for ACs derived from miscanthus and switchgrass are comparable with those of other lignocellulosic materials obtained using the same activation methods (Table 7). The only exceptions to that rule are clearly higher S_BET_ values for ACs produced by KOH activation from walnut shells and plum stones composed of sclereid cells with strongly lignified and thickened cell walls.

The results of TG analysis based on changes in the mass of respective samples at consecutive stages of thermolysis (Table 6) indicate varied thermal properties of the obtained ACs. Depending on the temperature, particular oxygen surface groups decompose. It is assumed that, in the range of 200–500 °C, stronger acidic groups (e.g., carboxyl) are degraded, whereas at 500–700 °C, weakly acidic groups (e.g., phenolic) disintegrate. However, at temperatures above 700 °C, groups of basic characters are broken down [56]. Therefore, the percentage share of functional groups in the AC structure corresponds to the thermal properties of the tested materials.

ACs obtained with the use of KOH as an activator contained more carboxyl groups of strongly acidic character, as well as a higher number of lactonic groups, which may be broken down and release carboxyl groups (Table 4). In the temperature range associated with the degradation of these groups (200–700 °C), the above-mentioned ACs showed greater mass losses as well as a significant effect of activator in the part of the range (Table 6). As shown above, the use of NaOH as the activator caused an increase in the number of basic oxygen groups (Table 4), which was also reflected in the results of TG analysis. These ACs showed greater mass losses and partially also a significant effect of activator (Table 6) in the temperature range of 700–1200 °C, i.e., specific to the decomposition of basic functional groups. For the tested ACs, mass losses were also determined in the whole range of thermal analysis, i.e., from 20 to 1200 °C. The values of these mass losses may be treated as a measure of the thermal stability of the obtained ACs. However, for particular precursors, the effect of the activators on thermal stability was generally opposite, which further confirms their different activation mechanisms. In addition, for most temperature ranges, ACs obtained by NaOH activation were slightly more stable. Overall, MG/NaOH, MSin/NaOH and MSac/KOH may be considered the most thermally resistant ACs. Nonetheless, it may be assumed that the above-mentioned ACs with the most developed porous structure, i.e., SG/NaOH and especially MSac/NaOH, have the highest potential applicability, e.g., in decontamination or purification, to replace carbons obtained so far from *M.* × *giganteus* [9,27].

## 5. Conclusions

Tested miscanthus species and switchgrass exhibited a biomass composition typical of the temperate climate and in part to its transitional or even continental subtype. These materials proved to be good feedstocks to produce activated carbons, comparable to traditional biomass sources. All obtained ACs were characterized by a rich chemical structure of the surface, a well-developed porous structure and sufficient thermal stability. The activators, KOH or NaOH, exerted a stronger effect on the AC surface as well as porosity parameters and thermal stability than biomass composition. Yet, the latter, particularly contents of lignin and mineral compounds, may have some impact on AC properties. This study has shown that the mechanisms of KOH and NaOH action on carbon precursors were not identical, which directly affected the structure and properties of carbon materials. Both activators mostly generated the formation of surface acidic functional groups, while it was at to larger extent in the case of KOH. However, more extensive specific surface areas, larger micropore volume, as well as slightly higher iodine numbers of ACs and thermal stability, were obtained when NaOH was used. This study provides a good basis for detailed studies on the structure and adsorption abilities of ACs obtained from miscanthus and switchgrass, as well as the effect of the composition of biomass harvested at different years and environments on AC properties. Nonetheless, this study has already demonstrated that ACs derived from *M. sacchariflorus* and to a lesser extent also those derived from switchgrass have surface and porosity parameters with the highest potential practical applicability. Hence, even if these crop species are not abundant effective biomass sources for energy purposes, they may become valuable feedstocks for activated carbons.

## Figures and Tables

**Table 1:** Lignocellulosic materials and derived activated carbons obtained in the study.

Lignocellulosic Material	Abbreviation	Activated Carbons
*M.* × *giganteus*	MG	MG/KOH, MG/NaOH
*M. sacchariflorus*	MSac	Msac/KOH, MSac/NaOH
*M. sinensis*	MSin	MSin/KOH, MSin/NaOH
*P. virgatum*-switchgrass	SG	SG/KOH, SG/NaOH

**Table 2:** Contents of main components of studied lignocellulosic raw materials. Significant differences between materials are marked by letter indexes, separately for each component.

Content (%)	MG	MSac	MSin	SG
Holocellulose	74.42 ^b^	73.68 ^b^	73.91 ^b^	70.77 ^a^
Cellulose	45.12 ^b^	44.57 ^b^	44.12 ^b^	40.30 ^a^
Hemicellulose	29.30 ^a^	29.11 ^a^	29.79 ^a^	30.47 ^a^
Pentosans	24.39 ^a^	24.71 ^a^	24.49 ^a^	24.35 ^a^
Lignin	22.21 ^c^	20.34 ^b^	19.52 ^a^	20.09 ^b^
Cellulose-Lignin	2.03 ^a^	2.19 ^b^	2.26 ^c^	2.01 ^a^

**Table 3:** Contents of extractives and ash in miscanthus and switchgrass lignocellulosic raw materials. Significant differences among materials are marked by letter indexes, separately for each substance.

	Substance (%):	MG	MSac	MSin	SG
Extracted in	Cold water	3.53 ^b^	2.61 ^a^	2.39 ^a^	7.12 ^c^
	Hot water	4.69 ^a^	5.41 ^b^	4.99 ^ab^	7.89 ^c^
	1% NaOH	32.70 ^a^	31.48 ^a^	33.45 ^b^	34.44 ^c^
	Organic solvents	2.54 ^a^	2.55 ^a^	2.97 ^ab^	3.14 ^c^
	Ash	2.63 ^b^	2.16 ^a^	2.54 ^b^	2.20 ^a^

**Table 4:** Contents of surface oxygen functional groups in activated carbons derived from miscanthus and switchgrass biomass. Significant differences between activated carbons (ACs) are marked by letter indexes, separately for each group (in columns). Significant differences between concentrations of total acidic vs. basic groups for a given AC are indicated by asterisk (in respective rows).

AC	Functional Groups (mmol/g)				
	Acidic			Acidic (Total)	Basic (Total)
	Carboxylic	Lactonic	Phenolic		
MG/KOH	0.25 ^d^	0.39 ^b^	0.96 ^e^	*1.60 ^e^	0.67 ^b^
MSac/KOH	0.29 ^d^	0.49 ^c^	0.71 ^bc^	*1.49 ^d^	0.68 ^b^
MSin/KOH	0.30 ^d^	0.49 ^c^	0.72 ^c^	*1.51 ^d^	0.58 ^a^
SG/KOH	0.25 ^d^	0.45 ^c^	0.90 ^de^	*1.60 ^e^	0.75 ^c^
MG/NaOH	0.19 ^c^	0.35 ^b^	0.64 ^b^	*1.18 ^b^	0.77 ^c^
MSac/NaOH	0.05 ^a^	0.34 ^b^	0.83 ^d^	*1.22 ^bc^	0.82 ^d^
MSin/NaOH	0.15 ^bc^	0.39 ^b^	0.54 ^a^	*1.08 ^a^	0.98 ^e^
SG/NaOH	0.10 ^ab^	0.24 ^a^	0.95 ^e^	*1.29 ^c^	0.98 ^e^

**Table 5:** Parameters of the porous structure in activated carbons derived from miscanthus and switchgrass lignocellulosic biomass.

AC	Surface Area (m^2^/g)	Pore Volume (cm^3^/g)			Pore Width (nm)	Iodine Number (mg/g)
	S_BET_	V_T_	V_micro_	V_meso_	d_av_	IN
MG/KOH	1542	1.05	0.53	0.52	2.72	1220
MSac/KOH	1396	1.05	0.50	0.55	3.01	1230
MSin/KOH	1400	0.98	0.45	0.53	2.80	1200
SG/KOH	1467	1.03	0.50	0.53	2.81	1210
MG/NaOH	1689	1.08	0.59	0.49	2.56	1210
MSac/NaOH	1796	1.52	0.62	0.90	3.38	1240
MSin/NaOH	1612	0.95	0.63	0.32	2.36	1220
SG/NaOH	1731	1.24	0.61	0.63	2.86	1230
p-value for effect of activator	0.003 *	0.219	0.001 *	0.684	0.852	0.315
p-value for effect of precursor	0.936	0.451	0.995	0.350	0.132	0.276

*—significant.

**Table 6:** Thermogravimetric analysis of activated carbons derived from miscanthus and switchgrass lignocellulosic raw materials.

ACs	Mass Loss (%)					
	20–200 °C	200–500 °C	500–700 °C	700–900 °C	900–1200 °C	20–1200 °C
MG/KOH	2.86	4.54	3.91	3.97	5.73	21.01
MSac/KOH	2.31	3.90	3.53	3.81	6.16	19.71
MSin/KOH	2.52	4.80	4.39	4.19	7.25	23.15
SG/KOH	2.42	4.06	3.75	3.67	6.26	20.15
MG/NaOH	2.52	3.51	2.36	3.12	6.22	17.73
MSac/NaOH	2.27	3.12	1.99	3.43	13.38	24.20
MSin/NaOH	2.14	3.74	2.38	2.79	8.16	19.21
SG/NaOH	2.07	4.74	2.37	3.28	11.02	23.47
p-value for effect of activator	0.123	0.224	<0.001 *	0.005 *	0.083	0.936
p-value for effect of precursor	0.298	0.573	0.957	0.994	0.678	0.766

*–significant.

**Table 7:** Parameters of porous structure of chemically activated carbons derived from different precursors.

Precursor	Activation Agent (°C)	Surface Area (m^2^/g)	Pore Volume (cm^3^/g)			Pore Diameter (nm)	Ref.
		S_BET_	V_T_	V_micro_	V_meso_	d_av_	
Walnut shells	KOH/750	2041	1.12	0.33	0.79	2.19	[53]
	NaOH/750	1864	1.09	0.29	0.80	2.34	
Pistachio shells	NaOH/750	1710	1.07	0.63	0.44	2.50	
Peanut shells	NaOH/750	1793	1.24	0.30	0.94	2.77	
Common osier stems	KOH/750	1810	1.11	0.31	0.80	2.45	[54]
Hemp stems	KOH/750	1625	1.08	0.53	0.55	2.66	
Flax stems	KOH/750	1507	1.01	0.51	0.50	2.68	
Plum stones	KOH/750	2270	1.09	1.02	0.07	1.92	
Hornbeam wood	KOH/950	1862	1.00	0.79	0.21	2.15	[55]
	NaOH/950	1492	0.98	0.25	0.73	2.61	
